# Comparative dosimetric study of radiotherapy in high-grade gliomas based on the guidelines of EORTC and NRG-2019 target delineation

**DOI:** 10.3389/fonc.2023.1108587

**Published:** 2023-05-23

**Authors:** Ouying Yan, Haibo Teng, Cuihong Jiang, Lili He, Shuai Xiao, Yanxian Li, Wenqiong Wu, Qi Zhao, Xu Ye, Wen Liu, Changgen Fan, Xiangwei Wu, Feng Liu

**Affiliations:** ^1^ Department of Radiation Oncology, Hunan Cancer Hospital and The Affiliated Cancer Hospital of Xiangya School of Medicine, Central South University, Changsha, China; ^2^ Department of Neurosurgery, West China Hospital, Sichuan University, Chengdu, China

**Keywords:** high grade gliomas, radiotherapy, target delineation, dosimetric, OARs

## Abstract

**Purpose:**

Radiotherapy is one of the most important treatments for high-grade glioma (HGG), but the best way to delineate the target areas for radiotherapy remains controversial, so our aim was to compare the dosimetric differences in radiation treatment plans generated based on the European Organization for Research and Treatment of Cancer (EORTC) and National Research Group (NRG) consensus to provide evidence for optimal target delineation for HGG.

**Methods:**

We prospectively enrolled 13 patients with a confirmed HGG from our hospital and assessed dosimetric differences in radiotherapy treatment plans generated according to the EORTC and NRG-2019 guidelines. For each patient, two treatment plans were generated. Dosimetric parameters were compared by dose–volume histograms for each plan.

**Results:**

The median volume for planning target volume (PTV) of EORTC plans, PTV1 of NRG-2019 plans, and PTV2 of NRG-2019 plans were 336.6 cm^3^ (range, 161.1–511.5 cm^3^), 365.3 cm^3^ (range, 123.4–535.0 cm^3^), and 263.2 cm^3^ (range, 116.8–497.7 cm^3^), respectively. Both treatment plans were found to have similar efficiency and evaluated as acceptable for patient treatment. Both treatment plans showed well conformal index and homogeneity index and were not statistically significantly different (P = 0.397 and P = 0.427, respectively). There was no significant difference in the volume percent of brain irradiated to 30, 46, and 60 Gy according to different target delineations (P = 0.397, P = 0.590, and P = 0.739, respectively). These two plans also showed no significant differences in the doses to the brain stem, optic chiasm, left and right optic nerves, left and right lens, left and right eyes, pituitary, and left and right temporal lobes (P = 0.858, P = 0.858, P = 0.701 and P = 0.794, P = 0.701 and P = 0.427, P = 0.489 and P = 0.898, P = 0.626, and P = 0.942 and P = 0.161, respectively).

**Conclusion:**

The NRG-2019 project did not increase the dose of organs at risk (OARs) radiation. This is a significant finding that further lays the groundwork for the application of the NRG-2019 consensus in the treatment of patients with HGGs.

**Clinical trial registration:**

The effect of radiotherapy target area and glial fibrillary acidic protein (GFAP) on the prognosis of high-grade glioma and its mechanism, number ChiCTR2100046667. Registered 26 May 2021.

## Introduction

Gliomas are the most prevalent malignancies of the central nervous system (CNS) (1), accounting for 30%–40% of all primary CNS tumors with a high incidence of recurrence and an extremely poor prognosis (2, 3). Gliomas are graded into grades I–IV according to the 2016 World Health Organization (WHO) classification system (4, 5). The grades III and IV are referred to as high-grade glioma (HGG), which has the most invasive growth pattern (6). The present standard of treatment for HGG is the combination of maximal surgical debulking, radiation therapy (RT), and temozolomide (TMZ) chemotherapy (7). RT can kill cancer cells effectively to delay or prevent its progression; however, the optimal volume for HGG target area delineation remains a controversial issue (8). Different guidelines recommend different target region delineation; the main point of the argument is whether the peritumoral edema was included.

The protocols of Radiation Therapy Oncology Group (RTOG) and European Organization for Research and Treatment of Cancer (EORTC) are the current commonly used methods of the target delineation of HGG gliomas (9–11). According to the RTOG present protocol, the initial clinical target volume (CTV) involves resection cavity, residual tumor, and postoperative peritumoral edema plus 2 cm, followed by a boost area defined as a resection cavity or residual tumor plus 2 cm and prescribed to 60 Gy based on RTOG 0525 and RTOG 8525 trials. In contrast, according to the delineation method of target area practiced at EORTC, the CTV is defined by the surgical field or residual tumor plus 2 cm without the involvement of peritumoral edema.

There are no unified guidelines for the delineation of glioma target area. The rationale for involving peritumoral edema in the CTV is that pathologically identified cancer cells have been observed in such areas in some studies (12). However, there would be a large brain volume irradiated to high dose in cases of severe edema, which may increase the dose of normal tissues and the potential radiation toxicity to some extent (8). Recently, in 2019, the National Research Group (NRG) consensus (referred to simply as the NRG-2019) was published, which proposed that the CTV of glioma should be trimmed along the anatomical structure based on the previous RTOG target delineation method, and the results showed that the volume of irradiated brain tissue would be decreased (13). This is an interesting phenomenon. On the basis of original RTOG guideline that includes peritumoral edema, NRG-2019 consensus further reduced the volume of target area, which will be of great clinical significance to compare with EORTC relatively small volumes principle without edema. At present, there is no target domain dosimetry comparison of glioma based on NRG-2019 and EORTC target delineation guidelines.

This article was designed to compare the differences of target delineation methods based on NRG-2019 and EORTC guidelines and to provide a theoretical basis for the application of the latest consensus of NRG-2019 target delineation, so that the radiotherapy efficacy of patients with HGG can be further boosted in the actual clinical practice.

## Methods

### Patient characteristics

A prospective study was performed in our center from May 2021 and December 2021 to assess the dosimetric differences in volumetric modulated arc therapy plans generated on the basis of the EORTC and NRG-2019 guidelines. Thirteen adult patients with HGG were included in this research. The inclusion criteria are as follows: (a) patients were diagnosed pathologically with WHO III-IV glioma; (b) all patients were greater than 18 years; (c) all patients underwent surgical resection, and KPS ≥ 70; (d) having preoperative contrast enhanced T1 and T2 MR images; and (e) all participants have signed the informed consent form. The exclusion criteria are as follows: (a) patients previously received pre-operative radiotherapy or chemotherapy; (b) patients had other serious systemic diseases or synchronous multiple primary malignancies; (c) patients with any active infection; and (d) women who were pregnant or breastfeeding. All of patients received RT with concomitant TMZ within 2–4 weeks after surgery, followed by adjuvant TMZ. All the patients need to conduct the simulation CT images for treatment planning, and, for each patient, two treatment plans were generated. Details of the patient characteristics are presented in **Table 1**.

**Table 1:** Clinical characteristics of 13 patients with high-grade glioma.

Parameter	N
Gender	
Male (n)	8 (61.5%)
Female (n)	5 (38.5%)
Age	
Median (year)	54
Range (year)	23–63
Side	
Right (n)	10 (77%)
Left (n)	3 (23%)
Preoperative Epilepsia	
Yes	3 (23%)
No	10 (77%)
Surgical type	
Total resection	13 (100%)
Subtotal resection	0 (0)
Tumor size (cm)	
Median	6
Range	3.8–8
IDH status	
Mutation	1 (7.6%)
Wild-type	12 (92.4%)
Tumor focality	
Unifocal	13 (100%)
Multifocal	0 (0)
Tumor location	
Frontal lobes(n)	4 (30.8%)
Temporal lobes(n)	4 (30.8%)
Occipital lobes (n)	1 (7.7%)
Fronto-temporal lobes (n)	2 (15.3%)
Fronto-parietal lobes (n)	1 (7.7%)
Parieto-temporal lobes (n)	1 (7.7%)

### Treatment planning

For positioning prior to radiotherapy, thermoplastic head masks were utilized to immobilize patients in the supine position and to ensure the subsequent reproducible positioning. CT scanning was performed acquired from the entire cranium with spiral mode in slices thickness of 2.5 mm, and, then, the CT images was transmitted to Monaco software for image fusion and delineation. All dose distribution and treatment plans were performed on the Eclipse treatment planning system (Varian Medical Systems). Two target areas were delineated for each of 13 patients according to NRG principle and EORTC principle, and treatment plans were created, respectively.

### Target definition and prescription dose

**Figure 1:**
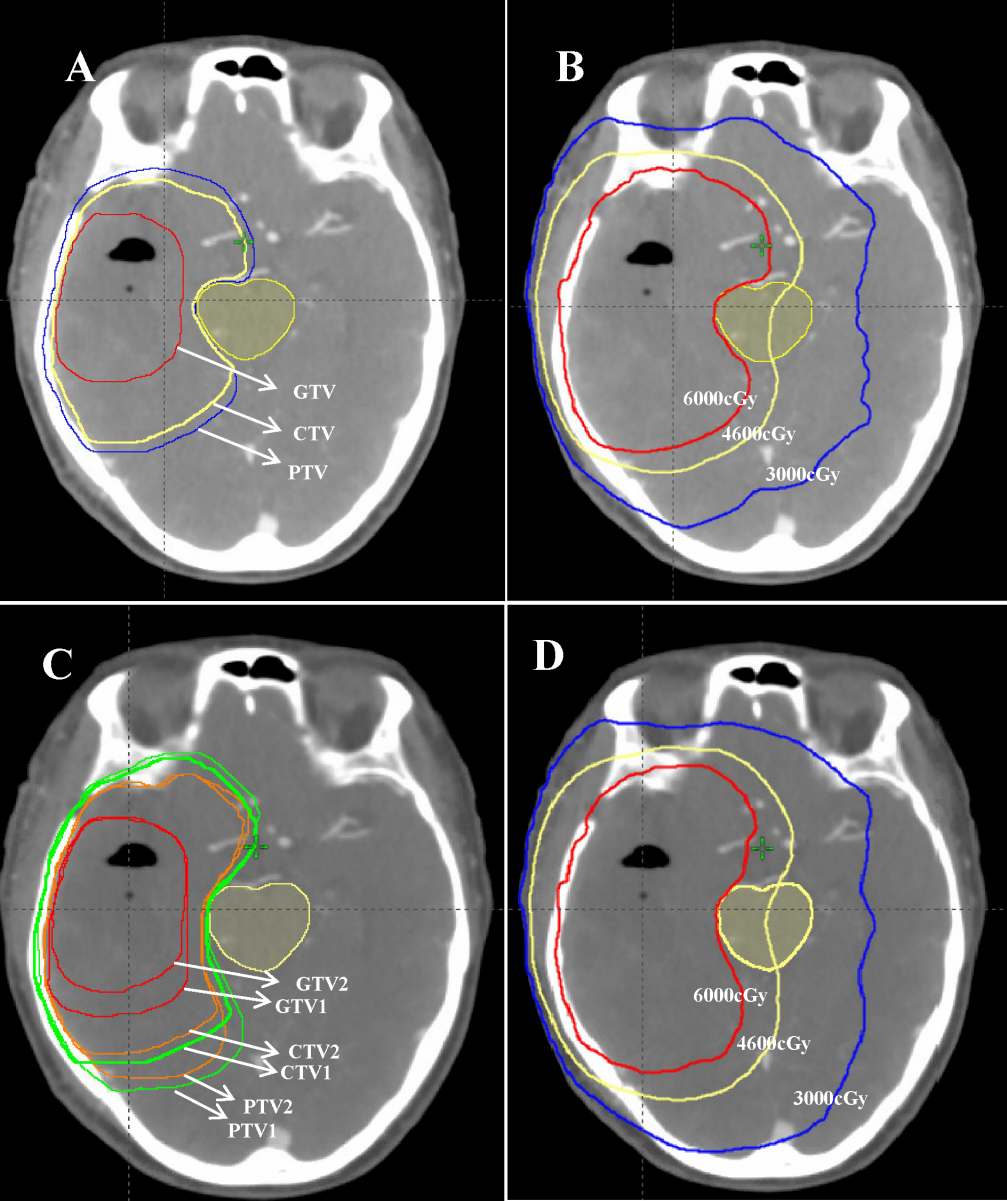
A 57-year-old female patient with postoperative pathology showing glioblastoma (WHO IV, 2016). **(A, C)** Target area delineation figures: **(A)** the EORTC delineation method and **(C)** the NRG-2019 delineation method. **(B, D)** Schematic illustrations of the planned dose distribution of EORTC and NRG-2019, respectively.

For the EORTC group, according to EORTC 26052-22053 guidelines (14): (1) gross tumor volume (GTV) refers to the MRI T1 enhancement area, resected cavity, and residual tumor excluding the peritumoral edema area; (2) CTV, formed by the 2-cm outward expansion of the GTV; and (3) planning target volume PTV, 0.3-cm outward expansion of CTV. The prescription dose was 95% PTV 60 Gy in 30 fractions. For the NRG-2019 group, according to the 2019 NRG expert consensus: (1) GTV1 refers to the T1 enhancement area, resected cavity, residual tumor, T2 FLAIR abnormal signal area, and peritumoral edema on postoperative MRI. (2) CTV1 is formed by 2 cm of GTV1 external expansion, and PTV1 is equal to 0.3 cm of CTV1 external expansion. The prescribed dose was 46Gy in 23 fractions. (3) GTV1 was reduced to include only the T1-enhanced area, resected cavity, and residual tumor on postoperative MRI to form GTV2. GTV2 was expanded by 2 cm to form CTV2, and PTV2 was formed by 0.3 cm of CTV2 expansion. The prescription dose was 14 Gy in 7 fractions. (4) Both CTV1 and CTV2 were further anatomically trimmed according to the anatomical structures of the cerebral falx, cerebellar curtain, and ventricles. The specific outlining method was referred to the NRG consensus guidelines published in 2019 (13). The target delineation diagram is shown in **Figure 1**, whereas the limits of organs at risk are shown in **Table 2**.

**Table 2:** Dose restriction on organs at risk.

OARs	Dose limits
Brain	Dmean < 4,000 cGy
Brain stem	Dmax < 6,000 cGy
Pituitary	Dmax < 5,400 cGy
Temporal lobe	V65 < 1%
Left eyes	Dmax < 5,000 cGy
Right eyes	Dmax < 5,000 cGy
Left lens	Dmax < 6,000 cGy
Right lens	Dmax < 6,000 cGy
Optic chiasm	Dmax < 6,000 cGy
Left optic nerve	Dmax < 5,400 cGy
Right optic nerve	Dmax < 5,400 cGy

### Treatment plans evaluation

The dose–volume histograms (DVHs) were calculated for each plan to compare the dosimetric parameters. The parameters—conformity index (CI), homogeneity index (HI), D95% (the dose received 95% volume of the PTV), D98%, D2%, D50%, and D5%—were quantified from the PTVs. The CI was calculated to assess the conformity degree of dose distribution to the target volume. The formula of CI was CI = PTV_PIV_/PTV × PTV_PIV_/PIV (PTV_PIV_, target volume encompassed within the prescription isodose; PIV, the reference isodose volume). The value of CI closer to 1 indicates the greater conformality. The HI was calculated to assess the dose uniformity in the target volume. The formula of HI was HI = (D2 − D98)/D50 (D2, D98, and D50 represent the corresponding dose covered 2%, 98%, and 50% of the target volume, respectively). The HI value closer to 0 demonstrates the better homogeneity within the PTV. The normal tissue containing the whole brain, brain stem, L/R optic nerves, optic chiasm, L/R eyes, L/R lens, and temporal lobe. Doses of the OARs and PTVs are all assessed by DVHs. To assess the volume of brain irradiated, V30, V46, and V60 were compared, where Vx represents the volume of irradiation above the designated dose.

### Statistical analysis

SPSS version 24.0 software was used to conduct the statistical analysis. The t-test and chi-square test was performed to compare the dosimetric differences among these two treatment plans formed by two different outlining methods for the 13 patients. P-values < 0.05 (two-tailed) were regarded as statistically significant.

## Results

### Patient characteristics

Thirteen patients with HGG proved by postoperative histopathology were prospectively enrolled in this study in 2021 (**Table 1**). As listed in **Table 1**, there were eight (61.5%) male and five (38.5%) female patients. All patients were ranged in age from 23 to 63 years old (median age, 54 years). The median volume for PTV of EORTC plans was 336.6 cm^3^ (range, 161.1–511.5 cm^3^), the median volume for PTV1 of NRG-2019 plans was 365.3 cm^3^ (range, 123.4–535.0 cm^3^), and the median volume for PTV2 of NRG-2019 plans was 263.2 cm^3^ (range, 116.8–497.7 cm^3^).

### PTV doses

As shown in **Table 3**, both plans showed well CI and HI and were not statistically significantly different in these two plans. All treatment plans were found to have similar efficiency and evaluated as acceptable for patient treatment. The D2%, D5%, D50%, D95%, and D98% were similar among EORTC and NRG-2019 plans (**Table 3**). However, the PTV1 of NRG-2019 plans was significantly larger than the PTV of EORTC plans.

**Table 3:** Comparison of PTVs dosimetric parameters.

Parameter	NRG-2019 _(PTV2)_		EORTC		Z	P
	Median	(25th, 75th percentiles)	Median	(25th, 75th percentiles)		
D2% (cGy)	63.45	63.28, 64.47	63.88	63.56, 64.41	−0.795	0.427
D5% (cGy)	63.18	63.05, 64.29	63.65	63.30, 64.09	−0.949	0.343
D50% (cGy)	62.54	61.97, 63.01	62.46	62.32, 62.69	−0.128	0.898
D95% (cGy)	60.98	60.86, 61.17	60.24	59.66, 60.49	−4.077	0.000
D98% (cGy)	60.76	59.83, 61.97	59.06	57.59, 59.60	−3.667	0.000
PTV volume (cm^3^)	263.2	214.74, 346.35	336.6	263.60, 404.40	−1.462	0.144
Homogeneity index	0.068	0.065, 0.078	0.074	0.049, 0.106	−0.795	0.397
Conformity index	0.933	0.91, 0.95	0.923	0.871, 0.952	−0.846	0.427

### Brain irradiated volume

The median whole brain volume was 1376.5 cm^3^ (range, 1,126.5–1,479.5 cm^3^). There was no significant difference in the volume percent of brain irradiated to 30, 46, and 60 Gy according to different target delineations (P = 0.397, P = 0.590, and P = 0.739, respectively, **Table 4**).

**Table 4:** Percent of volume of brain irradiated according to different plans.

Normal Brain	NRG-2019 _(PTV2)_		EORTC		Z	P
	Median	(25th, 75th percentiles)	Median	(25th, 75th percentiles)		
Dmean (cGy)	35.18	27.08, 39.02	33.02	25.11, 35.62	−1.103	0.270
Dmax (cGy)	65.61	64.61, 66.47	65.74	64.85, 66.23	−0.231	0.817
Dmin (cGy)	1.47	0.64, 1.81	1.25	0.55, 1.63	−0.641	0.512
V30 (cc)	745.52	573.74, 844.33	677.19	480.68, 747.64	−0.846	0.397
V46 (cc)	485.15	354.28, 536.32	453.46	333.88, 488.52	−0.538	0.590
V60 (cc)	296.56	248.45, 340.83	304.23	248.45, 344.40	−0.333	0.739
Volume (cc)	1381.1	1335.6, 1436.6	1381.1	1335.6, 1436.6	,	,

### OAR doses

The doses of normal organs were all within the clinically acceptable limits. These two plans also showed no significant differences in the doses to the brain stem, optic chiasm, left and right optic nerves, left and right lens, left and right eyes, pituitary, and left and right temporal lobes (P = 0.858, P = 0.858, P = 0.701 and P = 0.794, P = 0.701 and P = 0.427, P = 0.489 and P = 0.898, P = 0.626, and P = 0.942 and P = 0.161, respectively; **Table 5**).

**Table 5:** Dosimetric comparison of NRG-2019 and EORTC for ORAs in 13 patients with HGG.

Variable	NRG-2019		EORTC		Z	P
	Median (Gy)	(25th, 75th percentiles)	Median (Gy)	(25th, 75th percentiles)		
Brain stem						
Dmean	28.82	(18.83, 31.89)	28.71	(24.08, 35.90)	−0.179	0.858
Dmax	60.11	(53.88, 61.36)	60.87	(55.92, 61.90)	−0.744	0.457
Dmin	2.08	(1.34, 2.69)	2.00	(1.61, 2.42)	−0.436	0.663
Optic chiasm						
Dmean	38.87	(21.52, 47.51)	41.85	(17.61, 50.44)	−0.179	0.858
Dmax	51.30	(27.10, 58.26)	53.90	(20.93, 61.03)	−0.333	0.739
Dmin	32.28	(15.63, 36.78)	26.34	(11.48, 37.66)	−0.385	0.701
Left optic nerve						
Dmean	19.94	(10.00, 23.21)	16.47	(8.04, 26.25)	−0.385	0.701
Dmax	26.59	(16.73, 32.35)	25.92	(11.09, 41.26)	−0.385	0.701
Dmin	13.87	(6.03, 15.13)	11.28	(4.88, 14.77)	−0.795	0.427
Right optic nerve						
Dmean	19.84	(9.01, 26.03)	21.18	(6.52, 30.43)	−0.261	0.794
Dmax	26.47	(10.89, 42.84)	27.27	(7.73, 53.68)	−0.492	0.622
Dmin	11.29	(5.91, 15.20)	12.30	(4.54, 16.46)	−0.261	0.794
Left lens						
Dmean	4.73	(3.70, 6.02)	5.45	(3.64, 5.72)	−0.385	0.701
Dmax	5.22	(4.25, 6.77)	6.01	(4.50, 6.65)	−0.128	0.898
Dmin	4.24	(3.38, 5.27)	4.75	(3.12, 5.20)	−0.179	0.858
Right lens						
Dmean	5.00	(3.47, 5.75)	4.98	(4.83, 5.74)	−0.795	0.427
Dmax	5.61	(4.43, 7.21)	6.39	(5.36, 6.80)	−0.385	0.701
Dmin	4.37	(2.85, 4.83)	4.49	(3.78, 4.88)	−0.231	0.817
Pituitary						
Dmean	34.81	(22.09, 43.21)	37.97	(17.00, 46.60)	−0.487	0.626
Dmax	40.92	(24.86, 49.60)	47.35	(19.98, 55.08)	−0.436	0.663
Dmin	27.75	(13.05, 38.50)	28.04	(13.43, 39.76)	−0.231	0.817
Left eye						
Dmean	9.62	(6.36, 12.03)	9.38	(5.07, 10.91)	−0.692	0.489
Dmax	20.58	(14.33, 26.34)	21.73	(10.58, 22.89)	−0.333	0.739
Dmin	3.63	(2.18, 3.92)	3.17	(2.44, 4.00)	−0.333	0.739
Right eye						
Dmean	8.52	(6.28, 12.06)	8.63	(5.51, 13.09)	−0.128	0.898
Dmax	18.09	(15.08, 30.39)	20.38	(9.51, 32.67)	−0.128	0.898
Dmin	3.63	(2.28, 3.85)	3.72	(2.04, 4.40)	−0.436	0.663
Left temporal lobe						
Dmean	21.78	(12.83, 27.68)	19.15	(8.15, 30.59)	−0.072	0.942
Dmax	40.22	(32.13, 43.80)	40.03	(33.57, 49.87)	−0.362	0.717
Dmin	9.96	(1.00, 11.49)	8.08	(0.98, 10.76)	−0.507	0.612
Right temporal lobe						
Dmean	22.13	(16.28, 31.54)	23.36	(11.27, 40.98)	−1.403	0.161
Dmax	62.82	(32.99, 64.61)	60.51	(33.44, 64.56)	−0.702	0.483
Dmin	5.01	(1.94, 8.29)	6.94	(1.64, 12.68)	−1.140	0.254

## Discussion

RT plays a pivotal role in the management of HGG. Most guidelines advocate radiotherapy for the treatment of postoperative HGG, but there is significant controversy surrounding the recommendations for target area delineation. Currently, the RTOG and EORTC guideline are the most commonly used to contour the target area for radiotherapy of newly diagnosed grade III and IV gliomas (15, 16). Nevertheless, it still unclear which kind of target delineation guideline is best for glioma. At present, the main controversy over the delineation of HGG targets is whether CTV should include peritumoral edema. Reulen et al. (17) state that the extent of peritumoral edema is not directly related to the size of the tumor, but to the degree of malignancy. The range of peritumoral edema is approximately the same for the same histological type gliomas, and the more malignant the tumor, the greater the extent of peritumoral edema (17). Therefore, accurate localization and CTV delineation are the key factors for getting better curative effects of patients with HGG. Burger et al. (12) confirmed that there was tumor cell infiltration in peritumoral edema. If peritumoral edema area was regarded as the actual range of tumor cell involvement, then it might enlarge the irradiated field and increase the doses of OARs to some extent when severe edema is present. Another study on target volume delineation of glioblastoma also demonstrated that excessive CTV1 does not reduce recurrence rates at the tumor field margins or in the field but increases brain damage (8). Dobelbower et al. (18) also suggested that, when the target area was delineated with reference to peritumoral edema, the enhancement lesions were expanded by 1 cm or less may be considered. In contrast, some reports propose that most tumor cells in HGGs are located in the enhancement area of T1-weighted images, and, sometimes, the tumor cells would infiltrate into the peritumoral edematous zone (19, 20). It has also been demonstrated that histologically confirmed tumor cells are only fully covered when the irradiated field includes an enhanced lesion and peritumoral edema with outward expansion to 3 cm (21).

RTOG protocol recommends that the edema area should be included in the target area, whereas EORTC does not emphasize that all peritumoral edema areas must be included in the clinical target area. Previous studies have shown that, for patients with peritumoral edema >75 cm^3^, a target area plan using CTV expanded by 2 cm (but not considering the edema zone) could significantly reduce the median volume of normal brain irradiated by 30, 46, and 50 Gy compared to a RTOG plan (8). Large retrospective analysis showed that the volume of brain irradiated at 46 and 60 Gy in EORTC plan is smaller than that in the RTOG, but there is no significant difference in recurrence outcomes (22). Moreover, large clinical trials comparing the prognosis of these two delineation principles (EORTC and RTOG) also did not find significant differences in progression-free survival (PFS) or overall survival (OS) (10, 23). Thus, these results further imply that smaller expansions of CTV may not negatively impact patient outcomes. Many researchers have advocated a smaller margin than the extension advocated by RTOG guidelines in attempt to minimize the toxicity of RT in CNS (22, 24–27). With the continuous updating of expert consensus, the newly published NRG-2019 consensus guidelines in May 2019 proposed that the volume of irradiated brain can be reduced by an average of 13.6% (8.7%–17.9%) after more detailed trimming of the glioma CTV along the anatomy during target area outlining (13). The NRG-2019 consensus further reduces the volume of the CTV on the basis of the RTOG guidelines, which includes edema, and it is of great clinical significance to compare it with the EORTC principle that does not include edema. However, there are currently no dosimetry comparison studies between these two target area delineation guidelines of both NRG-2019 and EORTC. Therefore, we aimed to explore which delineation method resulted in the lower absorbed dose in the OARs and smaller irradiated volume of brain and to provide a stronger theoretical basis for the clinical application of this newly proposed NRG-2019 outline consensus in patients with HGG.

Our results show that the volume of PTV1 in the NRG-2019 group was significantly larger than that in the EORTC group. Similar to previous studies showing that the volume of the target area in the RTOG plan was larger than that in the EORTC plan. In our study, the NRG-2019 group target area outlining was further trimmed along anatomical pathways based on the RTOG outlining method. The results showed that, although it was possible to reduce the volume of irradiated brain compared to previous data, the target area volume was still significantly larger than that of the EORTC group.

It has been previously suggested that, when significant edema is present around the tumor, outlining the edematous area within the target area will somewhat enlarge the irradiation field and the organ-threatening exposure. In contrast, our results show that the anatomically modified NRG-2019 plan is not statistically significantly different from the EORTC plan in terms of organ-threatening irradiation exposure. In other words, in future clinical applications, clinicians can perform RT for HGGs that includes anatomically trimmed field irradiation of the edema band, depending on the patient’s actual situation. This allows for a degree of balance between irradiation of occult residual tumor in the edema area and the irradiated dose to the patient’s normal tissue. This can be used as an important reference in the actual radiotherapy work-up.

However, there are certain limitations to our study. As this is a prospective study, it is also still enrolling patients, and, therefore, the number of cases is now low. It is also the case that our study has not yet covered the prognosis of patients applying the two different plans, which will need to be followed up in our future clinical trials.

## Conclusion

Compared with EORTC guidelines for postoperative radiotherapy for HGG, the NRG-2019 project did not increase the dose of OARs radiation. This study further provides a foundation for the application of the NRG-2019 consensus in radiotherapy for patients with HGGs.

## Data availability statement

The raw data supporting the conclusions of this article will be made available by the authors, without undue reservation.

## Ethics statement

The study was approved by the Ethics Review Committee of Hunan Cancer Hospital/The Affiliated Cancer Hospital of Xiangya School of Medicine, Central South University (number for the approval: SBQLL-2021-003). All patients have provided written informed consent before participating in this study. The patients/participants provided their written informed consent to participate in this study.

## Author contributions

All the authors have contributed in the design, data collection, data analysis, manuscript writing and have read and approved the final version of the manuscript.
